# mtDNA release promotes cGAS-STING activation and accelerated aging of postmitotic muscle cells

**DOI:** 10.1038/s41419-024-06863-8

**Published:** 2024-07-23

**Authors:** Ying Li, Jie Cui, Lei Liu, William S. Hambright, Yutai Gan, Yajun Zhang, Shifeng Ren, Xianlin Yue, Liwei Shao, Yan Cui, Johnny Huard, Yanling Mu, Qingqiang Yao, Xiaodong Mu

**Affiliations:** 1 School of Pharmaceutical Sciences & Institute of Materia Medica, Shandong First Medical University & Shandong Academy of Medical Sciences, State Key Laboratory of Advanced Drug Delivery and Release Systems, Key Lab for Rare & Uncommon Diseases of Shandong Province, Jinan, Shandong China; 2 Center for Regenerative Sports Medicine, Steadman Philippon Research Institute, Vail, CO USA; 3 School of Medicine and Pharmacy, Ocean University of China, Qingdao, Shandong China; 4 Department of Orthopaedic Surgery, University of Texas Health Science Center, Houston, TX USA

**Keywords:** Senescence, Innate immunity, Mitochondria

## Abstract

The mechanism regulating cellular senescence of postmitotic muscle cells is still unknown. cGAS-STING innate immune signaling was found to mediate cellular senescence in various types of cells, including postmitotic neuron cells, which however has not been explored in postmitotic muscle cells. Here by studying the myofibers from Zmpste24^−/−^ progeria aged mice [an established mice model for Hutchinson-Gilford progeria syndrome (HGPS)], we observed senescence-associated phenotypes in Zmpste24^−/−^ myofibers, which is coupled with increased oxidative damage to mitochondrial DNA (mtDNA) and secretion of senescence-associated secretory phenotype (SASP) factors. Also, Zmpste24^−/−^ myofibers feature increased release of mtDNA from damaged mitochondria, mitophagy dysfunction, and activation of cGAS-STING. Meanwhile, increased mtDNA release in Zmpste24^−/−^ myofibers appeared to be related with increased VDAC1 oligomerization. Further, the inhibition of VDAC1 oligomerization in Zmpste24^−/−^ myofibers with VBIT4 reduced mtDNA release, cGAS-STING activation, and the expression of SASP factors. Our results reveal a novel mechanism of innate immune activation-associated cellular senescence in postmitotic muscle cells in aged muscle, which may help identify novel sets of diagnostic markers and therapeutic targets for progeria aging and aging-associated muscle diseases.

## Introduction

Skeletal muscle myofibers are terminally differentiated functional muscle cells, and repair of damaged myofibers in regenerating muscles is mainly achieved by the fusion of muscle stem cells with myofibers. Myofiber atrophy is one of the main defective phenotypes observed in both normally aged human and patients of progeria aging disease [1, 2]. The impaired muscle regeneration capacity during aging is largely caused by the progressive senescence and exhaustion of muscle stem cells [3], and senescence of muscle stem cells is closely associated with increased damage of nuclear DNA (ncDNA) [4]. However, since DNA in the nuclei of postmitotic myofibers does not bear the replication stress and risk of telomere shortening as muscle stem cells do, the potential molecular regulatory mechanism of myofiber aging is still unclear. In contrast to other muscle cells, myofibers are functional cells for muscle contraction, and are highly active in cellular metabolism and energy production. Mitochondria are responsible for energy production, and high energy consumption of myofibers is therefore supposed to generate more stress to mitochondria function [5], which may lead to higher level of mitochondrial damage. Damage to mitochondria often results in activation of mitophagy, an autophagic process specific for mitochondria, while impaired function of mitophagy can cause the accumulation of unprocessed damaged mitochondria and lead to cellular senescence [6]. Therefore, it is possible that increased mitochondrial damage and impaired mitophagy function could be a critical cause of myofiber defect in progeria aging.

Recently, cytoplasmic chromatin fragments (CCF) or cytoplasmic nuclear DNA (ncDNA) derived from the leaky nucleus were shown to trigger innate immune and cellular senescence through activation of cGAS-Sting signaling [7–11]. Also, innate immune response can be activated by the cytosolic danger-associated molecular patterns (DAMPs; i.e., mtROS and mtDNA) released from damaged mitochondria [10, 12]. Damaged mitochondria in healthy cells can be efficiently cleared by mitophagy, whereas impaired mitophagy function causes the accumulation of damaged mitochondria and subsequent chronic activation of innate immune response (i.e., cGAS, TLR9, and NLRP3 inflammasome signaling pathways) [13, 14]. Interestingly, a recent study just revealed that cGAS-Sting can be activated by mtDNA released from stressed mitochondria during aging, which is mediated by increased mitochondrial outer membrane permeabilization (MOMP) [15].

Hutchinson-Gilford progeria syndrome (HGPS) is an autosomal dominant disease associated with premature aging (progeria), leading to early death in childhood most often due to stroke or myocardial infarction [16–18]. Among various aging-related symptoms, severe muscle atrophy is also developed in HGPS patients [16–18]. Instead of Lamin A, high level of progerin is produced in HGPS cells and cause profound damage to normal nuclear structure and function [16–18]. Zmpste24 is Lamin A-processing zinc metalloproteinase required for cleaving the carboxylic group of prelamin A to Lamin A. Knocking out *Zmpste24* in mice (Z24^−/−^ mice) leads to accelerated aging and aging-related pathologies common to HGPS, and this has become an important murine model for HGPS and progeria [19–21]. Because progerin production becomes higher in cells of both accelerated aging and normal aging [22], the study of progeria Z24^−/−^ mice model is helpful to understand the mechanism of cellular senescence in both accelerated aging and normal aging.

cGAS-Sting signaling has been one of the most well-proven innate immune pathways to be involved in cellular senescence [7, 11, 23]. Although our recent study of Z24^−/−^ mice revealed the critical role of cGAS-Sting innate immune pathway in mediating the senescence of progeria muscle stem cells [24], whether innate immune activation also plays a role in mediating atrophic phenotypes of progeria aged myofibers is not known. Here we propose that increased mitochondrial damage and impaired mitophagy function could possibly promote the activation of innate immune signaling and subsequent cellular senescence in progeria-aged myofibers.

Our recent studies of progeria aging mice models revealed that the accelerated senescence of muscle stem cells is mainly induced by the elevated pro-inflammatory signaling, including ncDNA damage-induced activation of NF-κB signaling [25, 26] or ncDNA leaking-induced activation of cGAS-Sting innate immune signaling [24]. ncDNA damage and leaking are closely associated with increased nuclear structural abnormalities in senescent cells, including nuclear blebbing and micronuclei formation [9, 24]. However, it is unclear whether such regulatory mechanism involving nuclear abnormalities and cytosolic ncDNA in muscle stem cells during progeria aging can also dominate in the myofibers, because myofibers contain multiple nuclei and the adverse consequence of increased DNA damage in some nuclei can be partially compensated by other normal nuclei. Based on the facts above, it is possible that the potential innate immune activation in myofibers, if there is any, is not mainly caused by the damage and leaking of ncDNA, but by that of mtDNA. Voltage-dependent anion channel 1 (VDAC1) is the most abundant protein localized at mitochondrial outer membrane [27], and VDAC1 oligomerization was found to increase the permeabilization of mitochondrial membrane and promote mtDNA release into cytosol [28]. Also, the inhibition of VDAC1 oligomerization was shown to reduce mitochondria damage and mtDNA release [28]. Therefore, we propose that progeria-aged myofibers may develop increased VDAC1 oligomerization, which leads to increased mtDNA release into cytosol and innate immune activation.

Therefore in this study, with in vitro cultured myofibers isolated from aged-matched WT and Z24^−/−^ mice and with the help of Airyscan Fast Confocal Microscope, we investigated what could be the potential mechanism of cellular senescence in progeria aged myofibers, whether there is increased innate immune activation induced by mitochondrial damage and mtDNA release into cytosol, and what can be the key reason for potential mtDNA release from mitochondria. Since VDAC1 oligomerization in damaged mitochondria was shown to promote mtDNA release [28], here we also investigated whether there is increased VDAC1 oligomerization in Z24^−/−^ myofibers and whether the treatment of Z24^−/−^ myofibers with VBIT4 (a specific inhibitor of VDAC1 oligomerization) [28] may possibly reduce mitochondria damage, mtDNA release and activation of innate immune signaling.

## Results

### Skeletal muscle myofibers of Z24^−/−^ mice develops increased mitochondrial DNA (mtDNA) damage, but not nuclear DNA (ncDNA) damage

**Fig. 1:**
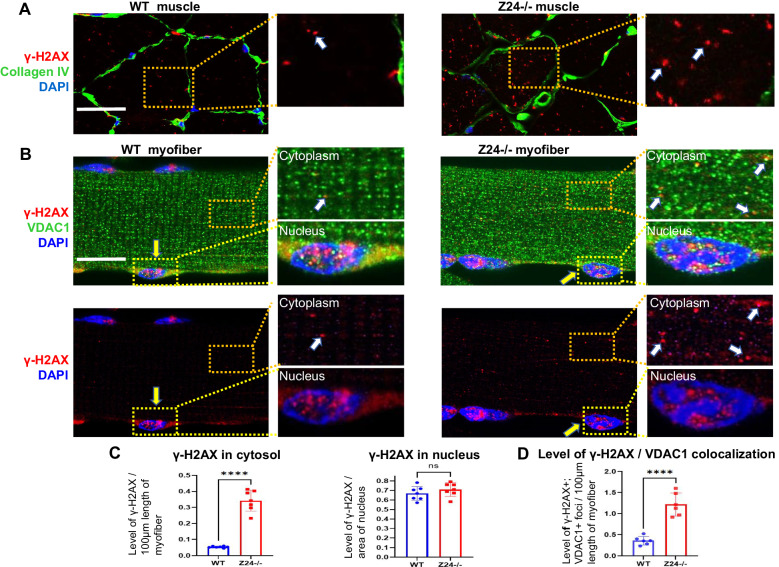
Z24^−/−^ myofibers display higher level of damage to mtDNA, but not ncDNA. **A** Immunostaining of skeletal muscle (gastrocnemius) cryosections of WT and Z24^−/−^ mice (5-month old, male) for γ-H2AX (red; a marker for DNA damage) and Collagen type IV (Collagen IV, a protein in extracellular matrix) in the myofibers in vivo, and observed with high-resolution Airyscan Fast Confocal Microscope. Immunostaining of Collagen VI serves to outline the edge of myofibers in vivo. Data for statistics is from muscle tissues of 6 mice. **B** Single myofibers isolated from FDB muscles of WT and Z24^−/−^ mice (5-month-old, male) were cultured in vitro and co-immunostained with antibodies to γ-H2AX and VDAC1 (a mitochondria membrane protein), and images of WT and Z24^−/−^ myofibers were measured and compared for signal intensity of γ-H2AX**+** foci within the same length of myofibers. It showed that, compared to WT myofiber, there is increased level of γ-H2AX+ foci in the cytoplasm of Z24^−/−^ myofibers (white arrows), but not in nucleus (yellow arrows). **C** Statistics of γ-H2AX in cytosol and nucleus of myofibers. Statistics are shown as mean values ± SD (*N* = 6). Scale bar: 50 μm (**A**), 25 μm (**B**).

Skeletal muscle (Gastrocnemius) was dissected from WT and Z24^−/−^ mice and immunofluorescent staining of γ-H2AX (a biomarker for DNA damage) and collagen type IV (an extracellular matrix protein) was performed with the cryosection slides of muscles. The result showed that, compared to myofibers in WT muscles, myofibers in Z24^−/−^ muscle developed increased level of γ-H2AX in the cytoplasm (Fig. 1A), indicating a potential increased level of mtDNA damage.

To verify that the damaged DNA in Z24^−/−^ myofibers is mtDNA, we isolated myofibers from Flexor digitorum brevis (FDB) muscles of the mice to culture as single myofibers in vitro, and performed co-immunofluorescent staining of γ-H2AX and VDAC1 (a mitochondria membrane protein). Result revealed a higher level of γ-H2AX deposition in Z24^−/−^ myofibers, with an increased level of γ-H2AX protein co-localizing with VDAC1 protein (Fig. 1B–D), verifying that there is increased damage of mtDNA in Z24^−/−^ myofibers. Importantly, the level of γ-H2AX in the nucleus showed no obvious difference between WT and Z24^−/−^ myofibers, suggesting that progeria-aged myofibers did not develop increased level of damage to ncDNA (Fig. 1B, C).

We also verified the status of DNA damage in nucleus of Z24^−/−^ myotubes formed from Muscle progenitor cells (MPCs) in vitro. MPCs were isolated from skeletal muscle (Gastrocnemius) of aged-matched WT mice and Z24^−/−^ mice (8-week old) according to the established preplate technique, which is generally based on slow-adhering properties of MPCs on collagen-coated cell culture surface [29–31]. MPCs were then cultured for 3 passages and plated for immunostaining assay of MyoD (a marker for myogenic cells), to verify their identity as a pure MPC population (Supplementary Fig. 1A). In the following, MPCs were cultured in myogenic differentiation medium to allow for the formation of multinucleated myotubes. Immunostaining of the cells with antibody against γ-H2AX further revealed that, in contract to the increased level of DNA damage in Z24^−/−^ mononucleated cells, nucleus in WT myotubes and Z24^−/−^ myotubes did not show significantly different level of DNA damage (Supplementary Fig. 1B). This result indicates that nucleus in multinucleated Z24^−/−^ myotubes generally do not develop more DNA damage than WT myotubes.

### Myofibers of Z24^−/−^ mice develops increased oxidative damage to mtDNA

**Fig. 2:**
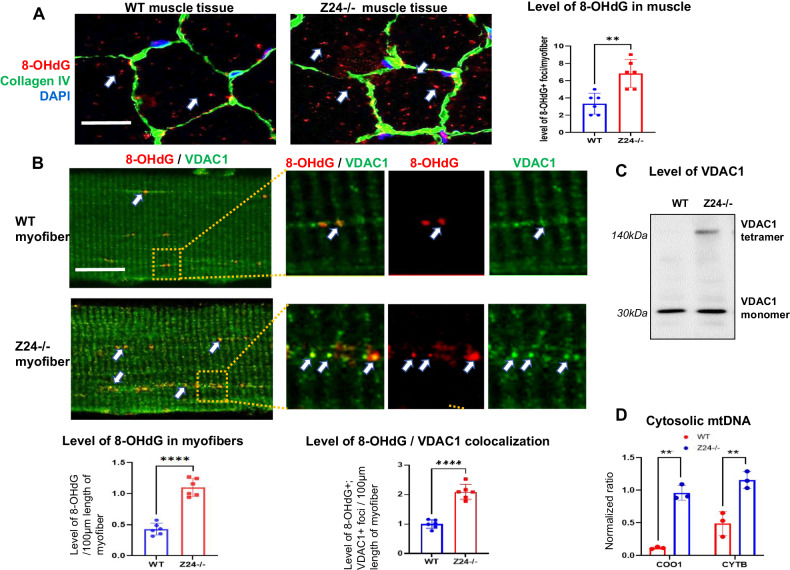
Z24^−/−^ myofibers display higher level of oxidative damage to mtDNA, mitochondrial VDAC1 oligomerization, and cytosolic mtDNA. **A** Immunostaining of skeletal muscle (gastrocnemius) cryosections of WT and Z24^−/−^ mice (5-month-old, male) revealed the differential level of 8-OHdG+ signal (red; a marker for oxidative DNA stress and damage) in the myofibers in vivo. Immunostaining of Collagen VI serves to outline the edge of myofibers in vivo. Data for statistics is from muscle tissues of 6 mice. **B** Single myofibers isolated from FDB muscles of WT and Z24^−/−^ mice (5-month-old, male) were cultured in vitro and co-immunostained with antibodies to 8-OHdG and VDAC1, and images of WT and Z24^−/−^ myofibers were measured and compared for signal intensity of 8-OHdG+ foci within the same length of myofibers. It showed increased level of 8-OHdG+ foci in Z24^−/−^ myofibers, which is frequently localized at VDAC1+ vesicles (white arrows). Data were from 3 groups of individual preparations of myofibers isolated from mice (5-month-old, male) (biological replicates). Statistics are shown as mean values ± SD (*N* = 6). **C** Western blot assay was performed to compare the level of VDAC1 monomer and tetramer (oligomerized form) in WT and Z24^−/−^ myofibers. **D** Measurement of the level of mtDNA released into myofibers, RT-PCR assay of CCO1 (cytochrome C oxidative subunit 1, a mtDNA gene) was performed with DNA isolated from mitochondria-free cytosol of WT and Z24^−/−^ myofibers, with 18 s rDNA serving as a control. Statistics are shown as mean values ± SD (*N* = 6). Scale bar: 50 μm (**A**), 25 μm (**B**).

Oxidative stress is known to play a leading role in promoting cellular senescence. 8-Hydroxydeoxyguanosine (8-OHdG) is a biomarker of oxidative stress and damage to DNA, which usually occurs at a high level to mtDNA [32]. Skeletal muscle (Gastrocnemius) was dissected from WT and Z24^−/−^ mice and immunofluorescent staining of 8-OHdG and Collagen type IV was performed with the cryosection slides. Result showed that, compared to myofibers in WT muscles, myofibers in Z24^−/−^ muscle developed increased level of 8-OHdG in the cytoplasm (Fig. 2A). In order to further verify the elevated level of oxidative stress and damage in Z24^−/−^ myofibers, we performed co-immunofluorescent staining of 8-OHdG and VDAC1 in myofibers isolated from FDB muscles. Results revealed that, compared to myofibers of WT mice, myofibers of Z24^−/−^ mice displayed higher level of 8-OHdG in the cytoplasm; also, the colocalization of 8-OHdG and VDAC1 was increased in myofibers of Z24^−/−^ mice (Fig. 2B). These observations suggest that there is increased oxidative stress and damage to mtDNA in Z24^−/−^ myofibers, which could be associated with increased mitochondria damage or mitophagy dysfunction.

### Myofibers from Z24^−/−^ mice feature increased VDAC1 oligomerization and increased release of mtDNA

As it is shown above, immunostaining of VDAC1 in myofibers showed that there may be more VDAC1+ foci with higher fluorescent signal intensity in Z24^−/−^ myofibers (Fig. 2B), suggesting a potential increased level of expression and/or oligomerization of VDAC1 protein on the mitochondria membrane. The result of western blot assay further revealed that, there is increased level of VDAC1 tetramer in Z24^−/−^ myofibers, verifying that there is higher level of VDAC1 oligomerization (Fig. 2C). We have also performed western blot assay to check the potential oligomerization of VDAC2 and VDAC3 proteins, and the results showed that, VDAC2, but not VDAC3, also developed increased oligomerization in Z24^−/−^ myofibers (Supplementary Fig. 2).

VDAC oligomerization is associated with increased mitochondria damage and can lead to higher permeability of mitochondria membrane, which then facilitates the release of mtDNA into cytosol [28]. To verify the potentially increased mtDNA releasing in Z24^−/−^ myofibers, immunostaining of myofibers was performed with antibodies against DNA and VDAC1. It showed that, in contract to regular mitochondrial localization of mtDNA (overlayed with VDAC1) in WT myofibers, some of mtDNA localized outside of mitochondria (not overlayed with VDAC1) in Z24^−/−^ myofibers, especially at the area of mitochondria with higher VDAC1 signal (Supplementary Fig. 3A), indicating a potentially increased amount of mtDNA releasing from damaged mitochondria.

To further verify this observation, total DNA was isolated from mitochondria-free cytosol fraction of WT and Z24^−/−^ myofibers, and the potential presence of mtDNA in the cytosol was determined with RT-PCR. RT-PCR result showed that, the level of CCO1 (Cytochrome C oxidative subunit 1, a mitochondria gene) and CYTB (cytochrome b, a mitochondria gene) DNA in the cytosol of Z24^−/−^ myofibers was significantly higher than WT myofibers (Fig. 2D). This observation reveals that increased VDAC1 oligomerization in mitochondria of Z24^−/−^ myofibers could have led to increased mtDNA release into cytosol. Here before performing PCR assay, we had also performed western blot assay of VDAC1 protein with lysates from whole cytosol fraction and mitochondria-free cytosol fraction, to verify the purity of the isolated mitochondria-free cytosol fraction (Supplementary Fig. 3B).

### Myofibers from Z24^−/−^ mice develop increased activation of mitophagy

**Fig. 3:**
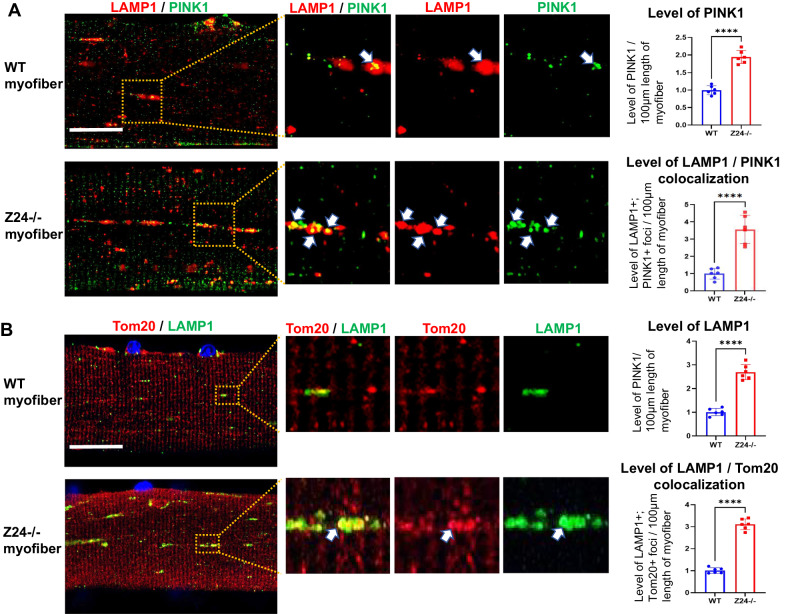
Z24^−/−^ myofibers develop higher level of mitophagy activation. **A** Single myofibers isolated from FDB muscles of WT and Z24^−/−^ mice (5-month old, male) were cultured in vitro and co-immunostained for LAMP1 and PINK1, and imaged with Airyscan Fast Confocal Microscope. It shows increased level of LAMP1+, PINK1+ and LAMP1/PINK1+ (white arrows) vesicles in Z24^−/−^ myofiber. **B** WT and Z24^−/−^ myofibers were co-immunostained for LAMP1 and Tom20, and it showed higher level of colocalization of the 2 proteins within the same vesicles (white arrows) in the Z24^−/−^ myofiber. DAPI staining serves to indicate the location of nuclei. Statistics are shown as mean values ± SD (*N* = 6). Scale bar: 25 μm (**A**, **B**).

In addition to increased oxidative stress, the increased mitochondrial damage observed in Z24^−/−^ myofibers may be also associated with impaired function of mitophagy, which is a specific process of removing damaged mitochondria by autophagy and prevents the accumulation of dysfunctional mitochondria to maintain cellular homeostasis [33]. PTEN-induced kinase 1 (PINK1) is a key protein involved in mitophagy modulation [33], and Lysosomal-associated membrane protein 1 (LAMP1, a marker for lysosome) is involved in the degradation process of mitophagy [34]. Thus we examined the potential status of mitophagy function in myofibers by observing the enrichment of PINK1 and LAMP1. The result showed that, compared to myofibers isolated from WT mice, myofibers from Z24^−/−^ mice showed elevated expression of LAMP1 and PINK1, while both of these 2 proteins were seen to specifically accumulate at some vesicles of same or adjacent locations in Z24^−/−^ myofibers (Fig. 3A). Similarly, co-immunostaining of Parkin (another key marker for mitophagy) and VDAC1 in myofibers further revealed that, there is also increased level of Parkin, and increased colocalization between Parkin and VDAC1 proteins in Z24^−/−^ myofibers (Supplementary Fig. 4). These results indicate that there may be higher level of mitophagy activation in Z24^−/−^ myofibers. Co-immunostaining of LAMP1 and Tom20 in WT and Z24^−/−^ myofibers was also performed and the result revealed increased colocalization of LAMP1 and Tom20, further indicating the close correlation between the activation of autophagic process and increased mitochondrial damage in Z24^−/−^ myofibers (Fig. 3B). It is noticed that the signals of LAMP1, PINK1 and Parkin are not homogenously “distributed” within the myofibers, but prone to accumulate and localize at some area. we think it is possibly a result of failed processing and clearance of damaged mitochondria by lysosome or mitophagy, causing the accumulation of dysfunctional mitophagy to form large vesicle-like structures.

### Myofibers from Z24^−/−^ mice develop increased dysfunction of mitophagy

**Fig. 4:**
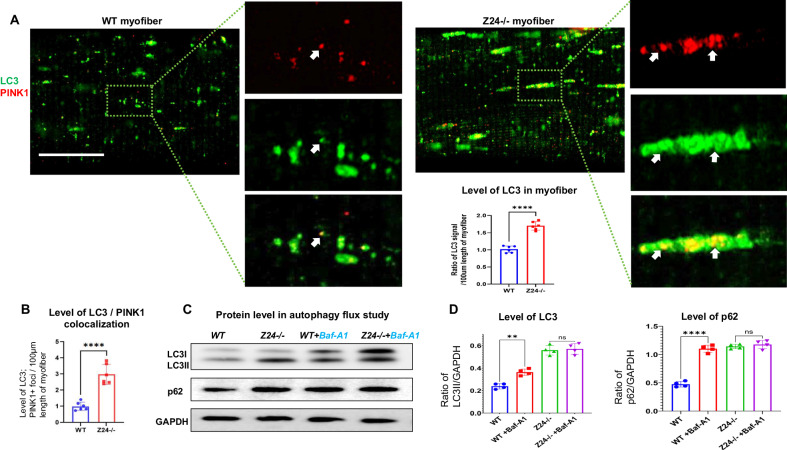
Z24^−/−^ myofibers develop higher level of autophagy initiation but increased mitophagy dysfunction. **A** Single myofibers isolated from FDB muscles of WT and Z24^−/−^ mice (5-month-old, male) were cultured in vitro and were co-immunostained for LC3 and PINK1, and imaged with Airyscan Fast Confocal Microscope. It shows increased level of LC3 and colocalization of LC3 with PINK1 in Z24^−/−^ myofiber. Arrows mark LC3+/PINK1+ vesicles. **B** Statistics of colocalization of LC3 and PINK1 in WT and Z24^−/−^ myofibers**. C** Western blot results of LC3 (I and II) and p62 proteins in WT and Z24^−/−^ myofibers from autophagy flux study. WT and Z24^−/−^ myofibers were treated with the lysosomal inhibitor Bafilomycin A1 (100 nM for 4 h). **D** Statistics of changes of LC3 and p62 in autophagy flux study. Statistics are shown as mean values ± SD (*N* = 6 or 4). Scale bar: 25 μm (**A**).

In addition to the increased activation or initiation of mitophagy, the significantly increased accumulation of mitophagy in Z24^−/−^ myofibers may also be a result of increased dysfunctional mitophagy that failed to move forward along the mitophagy process. To verify this possibility, we further examined the level of LC3 protein (a marker for autophagy initiation) and measuring the autophagy flux in myofibers with a lysosomal inhibitor Bafilomycin A1. Immunostaining of LC3 in WT and Z24^−/−^ myofibers showed that there was higher level of LC3 expression in Z24^−/−^ myofibers, indicating increased autophagy initiation in the cells (Fig. 4A, B). Western blot results of autophagy flux experiment showed that, the level of LC3II and p62 was increased in Z24^−/−^ myofiber compared to WT myofiber, verifying an increased basal level of autophagy activity, or autophagy initiation in Z24^−/−^ myofiber (Fig. 4C, D); however, the level of p62 and LC3II showed no significant difference in Z24^−/−^ myofiber with or without Bafilomycin A1 treatment, and the ratio of LC3II/LC3I was decreased, which indicates that the function of autophagy and mitophagy in Z24^−/−^ myofiber was possibly impaired (Fig. 4C, D). Therefore, it shows that there may be increased dysfunction of mitophagy in progeria-aged myofibers.

### Myofibers from Z24^−/−^ mice develops increased activation of cGAS-Sting signaling, which is associated with increased mtDNA releasing and mitophagy dysfunction

**Fig. 5:**
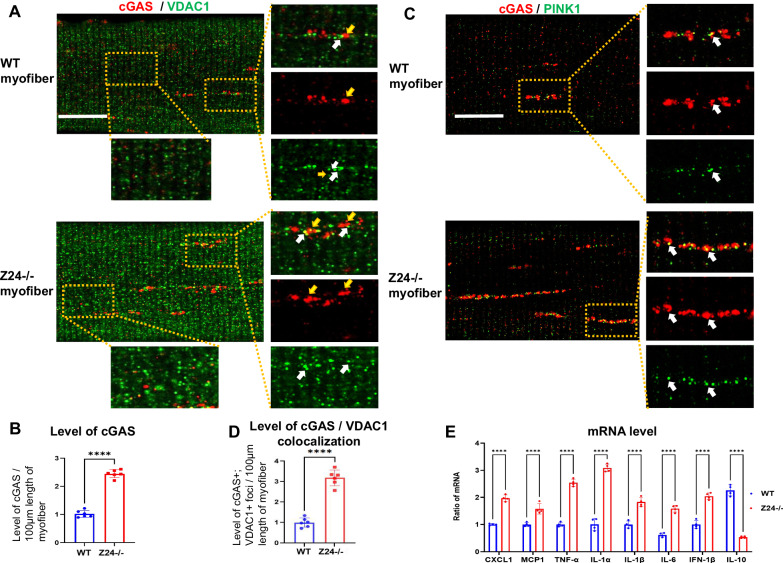
Z24^−/−^ myofibers develop higher level of innate immune (cGAS signaling) activation. **A** Single myofibers isolated from FDB muscles of WT and Z24^−/−^ mice (5-month-old, male) were cultured in vitro and were co-immunostained for cGAS and VDAC1, and imaged with Airyscan Fast Confocal Microscope. It shows increased level of cGAS+ vesciles in Z24^−/−^ myofiber, and cGAS frequently localized at adjacent locations to VDAC1-high vesicles (mitochondria with higher VDAC1+ signal than regular mitochondria) (yellow and white arrows), suggesting a potential accumulation of cGAS at location of damaged mitochondria (or mitophagy). **B** WT and Z24^−/−^ myofibers were co-immunostained with antibodies to cGAS and PINK1, and it showed higher level of colocalization of the 2 proteins in the adjacent or same (white arrows) vesicles in Z24^−/−^ myofibers. **C** Statistics of cGAS level in WT and Z24^−/−^ myofibers. **D** Statistics of colocalization of GAS and VDAC1 in WT and Z24^−/−^ myofibers**. E** Real-time-PCR assessment revealing the up-regulated expression of SASP factors in Z24^−/−^ myofibers. Each result was normalized to GAPDH as an endogenous control. The results are ratios of mRNA levels in Z24^−/−^ myofibers to those in the WT myofiber group, with average values in the WT myofiber group set as 1.0. Data were from 3 groups of individual preparations of myofibers isolated from mice (5-month-old, male) (biological replicates). And among each group of myofibers, single myofibers from 2 individual wells were counted as technical replicates. Statistics are shown as mean values ± SD (*N* = 6 or 4). Scale bar: 25 μm (**A**, **C**).

The presence of mtDNA in the cytosol is a part of a Danger-Associated Molecular Patterns (DAMPs) and can be recognized by the cyclic GMP-AMP synthase (cGAS) stimulator of interferon genes (Sting) pathway to activate innate immune response [10, 12]. The higher level of mtDNA release from damaged mitochondria observed in Z24^−/−^ myofiber is supposed to result in the activation of cGAS-Sting innate immune signaling. Co-immunostaining of cGAS and VDAC1 showed that Z24^−/−^ myofibers feature higher level of cGAS protein (Fig. 5A, B), which appears to accumulate largely around those mitochondria with higher VDAC1 signal. This observation suggests that cGAS may have been mobilized to the damaged mitochondria with increased VDAC1 oligomerization and mtDNA releasing.

Previous studies also reported interaction of cGAS with mitophagy in regulating innate immune activation [13]. Here, co-immunostaining of cGAS and PINK1 further verified that cGAS and PINK1 protein accumulated extensively at the same or adjacent location (Fig. 5C), indicating the potential close correlation between dysfunctional mitophagy process and innate immune activation.

### Myofibers from Z24^−/−^ mice express higher level of SASP (Senescence-Associated Secretary Phenotypes) factors

cGAS-Sting activation has been well proven to promote the senescence of various types of cells [7, 11, 23]. Senescent cells are known to feature increased expression of SASP factors. Here, RT-PCR assay of myofibers from WT and Z24^−/−^ myofibers were performed to verify the potential accelerated senescence in Z24^−/−^ myofibers. Result revealed that, Z24^−/−^ myofibers express higher level of SASP factors (i.e., TNF-α, IL-1α, IL-1β, IL-6, CXCL1, MCP1, IFN-1β, et. al.) (Fig. 5D), suggesting a senescence-associated phenotype being developed in Z24^−/−^ myofibers.

### Myofibers from Z24^−/−^ mice display higher level of NF-κB activation

**Fig. 6:**
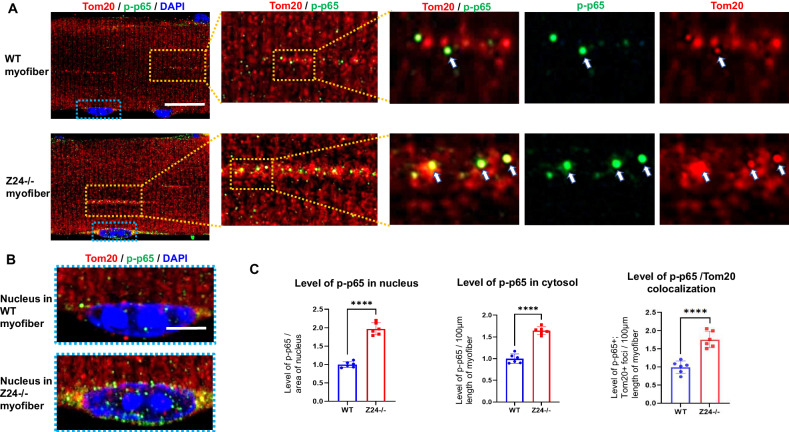
Z24^−/−^ myofibers develop higher level of NF-κB activation, with p-p65 being elevated in both nucleus and mitochondria. **A** Single myofibers isolated from FDB muscles of WT and Z24^−/−^ mice (5-month old, male) were cultured in vitro and were co-immunostained for Tom20 and p-p65, and were imaged with Airyscan Fast Confocal Microscope. It shows increased level of p-p65+ foci in the cytosol of Z24^−/−^ myofiber. Much of p-p65 protein co-localize with Tom20 protein, indicating a potential localization inside mitochondria. **B** Enlargement of nucleus images outlined with blue in **A**, p-p65 in the nucleus of WT and Z24^−/−^ myofibers is shown. **C** Statistics of p-p65 level in the cytosol or nuclei of WT and Z24^−/−^ myofibers, and the colocalization of p-p65 and Tom20. Statistics are shown as mean values ± SD (*N* = 6). Scale bar: 25 μm (A), 5 μm (**B**).

NF-κB is a crucial mediator of pro-inflammatory signaling and is greatly involved in the process of cellular senescence [25, 26]; also, NF-κB plays a critical role in the development of innate immunity by synergizing with IRF3 (a cGAS-Sting pathway factor) to induce high levels of type I IFNs [35, 36]. Immunostaining of phospho-p65 (p-p65, a subunit of NF-κB) in myofibers revealed that, Z24^−/−^ myofibers displayed higher level of p-p65 deposition in both cytoplasm and nucleus (Fig. 6A–C), further verifying the activation of innate immune signaling in Z24^−/−^ myofibers. Meanwhile, a fraction of p-p65 protein appeared to have translocated into mitochondria (Fig. 6A), which is able to cause impaired function of mtDNA and possibly associated with increased mitochondria damage [37].

### Inhibition of VDAC1 oligomerization in Z24^−/−^ myofibers represses activation of cGAS-Sting signaling and expression of SASP factors

**Fig. 7:**
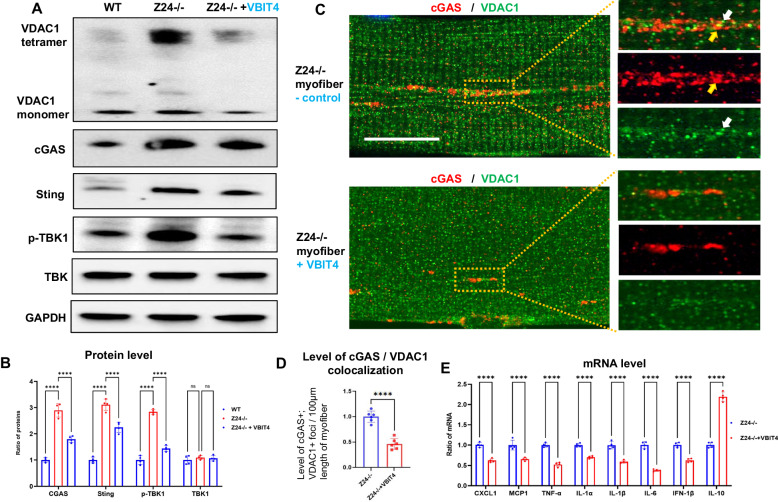
Inhibition of VDAC1 oligomerization reduces activation of cGAS-Sting and expression of SASP factors. **A** Western blot assay showing the reduced protein level of cGAS, Sting, p-TBK1 in Z24^−/−^ myofibers treated with VBIT4 (10 µM for 48 h). **B** Single myofibers isolated from FDB muscles Z24^−/−^ mice (5-month-old, male) were cultured in vitro. Z24^−/−^ myofibers with and without VBIT4 treatment were co-immunostained for cGAS and VDAC1, and imaged with Airyscan Fast Confocal Microscope. It shows reduced level of cGAS in VBIT4-treated Z24^−/−^ myofibers. Yellow arrow: cGAS+ vesicle; white arrow: VDAC1+ vesicle. **C** Real-time-PCR assessment showing the down-regulated expression of SASP factors in VBIT4-treated Z24^−/−^ myofibers. Each result was normalized to GAPDH as an endogenous control. The results are ratios of mRNA levels in VBIT4-treated Z24^−/−^ myofibers to those in the non-treated control group. **D** Statistics of colocalization of cGAS and VDAC1 in myofibers with or without VBIT4 treatment. **E** RT-PCR assay of expression level of SASP factors. Statistics are shown as mean values ± SD (*N* = 6). Scale bar: 25 μm (**C**).

Recent study showed that a specific inhibitor of VDAC1 oligomerization, VBIT4, is able to inhibit the oligomerization of VDAC1 and mtDNA release [28]. We treated Z24^−/−^ myofibers with VBIT4 for 48 h and observed significantly reduced level of VDAC1 oligomerization *via* western blot assay (Fig. 7A). Western blot assay also showed the significantly reduced level of cGAS-Sting signaling factors, including cGAS, Sting, and p-TBK1 (Fig. 7A). Immunostaining of cGAS also displayed reduced level of cGAS expression and accumulation in VBIT4-treated Z24^−/−^ myofibers (Fig. 7B). Meanwhile, RT-PCR assay further showed that the expression of SASP factors was significantly down-regulated in VBIT4-treated Z24^−/−^ myofibers (Fig. 7C). These observations indicate that the inhibition of VDAC1 oligomerization is effective in rescuing the defective senescence-associated phenotypes in Z24^−/−^ myofibers via reducing mtDNA-induced activation of cGAS-Sting signaling.

## Methods and materials

### Animal models

Zmpste24^−/−^ (Z24^−/−^) mice are deficient in Zmpste24, a metalloproteinase involved in the formation of mature lamin A, and are an established model for HGPS disease and premature aging [19, 20]. Age-matched Zmpste24^+/+^ (Z24^+/+^) littermates generated from the crossing of Zmpste24^+/^^−^ mice were used as wild-type (WT) controls. All the mice were maintained in the Center for Laboratory Animal Medicine and Care (CLAMC) at UTHealth (University of Texas Health Science Center at Houston) in accordance with established guidelines and protocols approved by the UTHealth Animal Welfare Committee. Male and female Z24^−/−^ mice display no obvious difference in progeria phenotypes, and both male and female mice were used for this study, with 6 male mice being studied for muscle and myofibers, and 6 female mice being studied for muscle stem/progenitor cells (MPCs).

### Isolation of myofibers and muscle stem/progenitor cells (MPCs)

Flexor digitorum brevis (FDB) muscles (a nearly homogeneous fast-twitch fiber type) from the skeletal muscle of Z24^−/−^ mice and WT mice (5-month-old, male) were surgically isolated and incubated in minimal essential media containing 0.1% gentamycin and 0.4% Collagenase A at 37 °C for 1.5–2.0 h, as previously described [29, 30]. To release single myofibers, FDB muscles were then triturated gently in serum containing media (MEM supplemented with 10% fetal bovine serum/FBS) without collagenase and incubated in 5% CO_2_ at 37 °C until used, typically 12–36 h later. Muscle stem/progenitor cells (MPCs) were isolated from the gastrocnemius muscle of Z24^−/−^ mice and WT mice (8-week-old, female) using the modified preplate technique [29, 30], based on their adhering capacity to collagen-coated surface/substrate. Mesenchymal stem cells (MSCs) and fibroblasts adhere quickly in hours and MPCs maintain floating in medium and only attached and start to grow days later [30, 31]. Once MPCs started to attach to the collagen-coated culture surface, MPCs were cultured in growth medium (DMEM supplemented with 20% FBS) and passed once they reached around 60% confluence. After culturing MPCs for 3 passages, their identity was verified by immunostaining of MyoD (a protein specifically expressed in myogenic cells), before being cultured in myogenic differentiation medium (DMEM supplemented with 2% FBS) and tested for the level of DNA damage (γ-H2AX + ) in the nucleus of MPCs or myotubes. MPCs were isolated from 8-week old Z24^−/−^ mice, because the amount of muscle stem cells become quickly exhausted after 2 months of age; and myofibers from FDB muscles were isolated from 5-month-old mice, since myofibers remain throughout the whole life span of Z24^−/−^ mice and it is the age displaying more severe progeria phenotypes than younger age [19, 20, 24].

### mRNA analysis via real time-PCR

total RNA was obtained from skeletal muscle myofibers of mice using the RNeasy Mini Kit (Qiagen, Inc., *Valencia*, CA) according to the manufacturer’s instructions. Reverse transcription was performed using an iScript cDNA Synthesis Kit (Bio-Rad., *Hercules*, CA). The sequences of PCR primers are given in Supplemental Table 1 for SASP genes (CXCL2, MCP1, TNFα, IL-1α, IL-1β, and IL-6) [38], IFN-β, IL-10, and GAPDH (glyceraldehyde 3-phosphate dehydrogenase). PCR reactions were performed using an iCycler thermal cycler (Bio-Rad). The cycling parameters used for all primers were as follows: 95 °C for 10 min; PCR, 40 cycles of 30 s at 95 °C for denaturation, 1 min at 54 °C for annealing, and 30 s at 72 °C for extension. All data were normalized to the expression of GAPDH.

### Western blotting

Cell lysates from enzymatically digested FDB myofibers were extracted with and quantified with the bicinchoninic acid (BCA) protein assay kit (Pierce, Rockford, IL), using BSA as standard. Cell lysates were heated at 100 °C for 10 min before loading for electrophoresis. Lysates were separated via SDS-PAGE and then transferred to polyvinyldifluoride (PVDF) membranes. Blots were incubated in blocking buffer (5%, w/v, dried skimmed milk in Tris-buffered saline, pH 7.4, and 0.2% Tween 20, TBST) followed by overnight incubation with appropriate antibodies diluted in blocking buffer (5% BSA in TBST). Antibodies for cGAS (Cell Signaling, *Danvers*, Massachusetts), Sting (Abcam, *Cambridge*, MA), p-TBK1 (Cell Signaling), TBK1 (Cell Signaling), LC3 (Abcam), p62 (Cell Signaling), VDACs (Abcam), and GAPDH (Santa Cruz, *Santa Cruz*, CA) were applied as proper concentration. More information about antibodies is included in Supplementary Table 2. Blots were then exposed to the Secondary Antibodies diluted in TBST for 60 min at room temperature and washed again. Original images of western blot are included in supplemental material. Specifically for the assay of VDAC oligomerization, we did not use DTT (DL-dithiothreitol) in the loading buffer to avoid breaking of covalent disulfide bond between oligomers of VDAC proteins; also, we heated the samples at 70 °C instead of 100 °C before loading for electrophoresis to avoid possible disruption of oligomers.

### Measurement of mtDNA level in the cytosol of myofibers

WT and Z24^−/−^ myofibers were collected for generating the fraction of mitochondria-free cytosol with the Mitochondria/Cytosol Fractionation Kit (Abcam). Protein from whole cytosol fraction and mitochondria-free cytosol fraction was loaded for western blot assay of VDAC1, to verify the purity of the mitochondria-free cytosol fraction. Total DNA was isolated from mitochondria-free cytosol, and the level of CCO1 (Cytochrome C oxidative subunit 1, a mitochondria/mt gene) and CYTB DNA in the cytosol was measured and compared between WT and Z24^−/−^myofibers with regular PCR, with 18S rDNA from nucleus serving as the reference gene control. Primers for CCO1 (mt gene) - Forward: 5′-GCCCCAGATATAGCATTCCC-3′; Reverse: 5′-GTTCATCCTGTTCCTGCTCC-3′; primers for CYTB (mt gene) - Forward: 5′-GCTTTCCACTTCATCTTACCATTTA-3′ Reverse: 5′-TGTTGGGTTGTTTGATCCTG-3′; primers for 18S rDNA (internal control)- Forward: 5′-TAGAGGGACAAGTGGCGTTC-3′; Reverse: 5′-CGCTGAGCCAGTCAGTGT-3′.

### Immunofluorescent staining and imaging

Cultured myofibers and MPCs were fixed with 4% paraformaldehyde, and frozen tissue sections were fixed with 10% formalin. The primary antibodies used – γ-H2AX (Cell Signaling), 8-OHdG (Santa Cruz), Collagen IV (Abcam), VDAC1 (Abcam), LAMP1 (Cell Signaling), PINK1 (Santa Cruz), Parkin (Santa Cruz), DNA (Abcam), Tom20 (Santa Cruz), LC3 (Abcam), cGAS (Cell Signaling), p-p65 (Cell Signaling) – were all applied at a 1:100 to 1:300 dilution. More information about antibodies is included in Supplementary Table 2. Cell nucleus was stained with DNA binding reagent 4′,6-diamidino-2-phenylindole (DAPI). Immunofluorescent images of myofibers were imaged and photographed with a Zeiss LSM980 with Airyscan Fast Confocal Microscope by standard Airyscan mode.

### VBIT4 inhibition of VDAC1 oligomerization

In order to find out the effect of VDAC1 oligomerization inhibition on Z24^−/−^ myofibers, 10 µM VBIT4 was applied to treat cultured myofibers for 48 h. The potential changes in the level of cGAS-Sting signaling factors (cGAS, Sting, p-TBK1 and TBK1) were then compared between myofibers with or without VBIT4 treatment by western blot or immunofluorescent staining.

### Autophagy flux assay

WT and Z24^−/−^ myofibers cultured in plates were treated with 100 nM Bafilomycin A1 for 4 h at 37 °C, or with an equivalent volume of sterile DMSO for 4 h, as the vehicle control. Cells were then lysed, and LC3 and p62 were analyzed using immunoblotting.

### Measurements of results and statistical analysis

Images captured with Airyscan Fast Confocal Microscope was analyzed using ZEN 2.3 imaging software (ZEISS Microscopy, Germany) and Image J software (version 1.32j; National Institutes of Health, Bethesda, MD,USA). Immunofluorescent staining images of myofibers were measured and compared for signal intensity or colocalization of proteins within the same length of myofibers (~100 µm length of myofiber) in different groups. Data from four or six samples from each subject or cell group (biological replicates) were pooled for statistical analysis. Results were processed with Graph Prism 8 and are given as the mean ± standard deviation (SD). The statistical significance of any difference was calculated using Student’s *t*-test or One-way Anova test. *p* values < 0.05 were considered statistically significant.

## Discussion

**Fig. 8:**
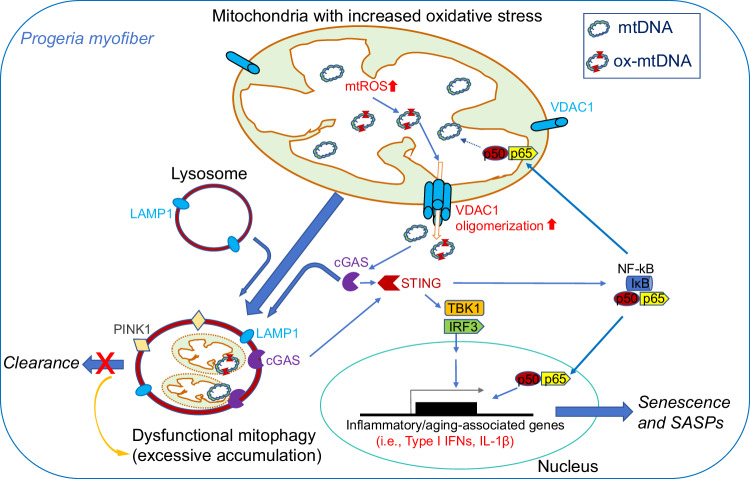
Schematic presentation of the potential mechanism of VDAC oligomerization-induced cGAS-Sting innate immune activation and cellular senescence-associated phenotype in progeria aged myofiber. Increased oxidative stress and Reactive oxygen species (ROS) production in the mitochondria of Z24^−/−^ myofiber may cause increased oxidative damage to mtDNA (ox-mtDNA), which promotes VDAC1 oligomerization and leaking of mtDNA into cytosol. cGAS-Sting innate immune signaling is activated by cytosolic mtDNA, leading to the activation of p-TBK1/IRF3 and NF-κB pathways. Both p-TBK1 and NF-κB activation promote the expression of pro-inflammatory factors, including type I IFNs. There is also potentially more NF-κB translocation into mitochondria, which negatively impacts the proper expression and function of mtDNA, leading to more mitochondria dysfunction and damage. Z24^−/−^ myofibers may also develop increased level of mitophagy initiation and dysfunction, leading to the accumulation of unprocessed damaged mitochondria and dysfunctional mitophagy, which is inductive of further cGAS-Sting innate immune activation.

Muscle atrophy is developed in various situations such as normal aging, progeria aging, cancer cachexia, and dystrophic muscle diseases. There have been great efforts in studying the role and mechanism of muscle stem cells in inducing muscle atrophy, but the exact molecular events inside atrophic myofibers are much less known. Our recent study of cells from HGPS patients and progeria mice model revealed that, the accelerated senescence of muscle stem cells is greatly associated with increased activation of cGAS-Sting innate immune signaling induced by nuclear DNA damage and leaking into cytosol [20]; however, the potential occurrence and mechanism of cellular senescence in postmitotic skeletal muscle myofibers still remains a mystery. Here by studying myofibers from WT and Z24^−/−^ progeria mice model, we have verified the senescence-associated phenotype in progeria-aged myofibers by finding out elevated expression of SASP factors, which is closely associated with increased activation of cGAS-Sting innate immune signaling; we also revealed that cGAS-Sting activation in progeria aged myofibers is possibly a result of increased mtDNA release into cytosol and mitophagy dysfunction, which is coupled with more oxidative damage to mtDNA and VDAC1 oligomerization; we further showed that specific inhibition of VDAC1 oligomerization in progeria aged myofibers was able to reduce the activation of cGAS-Sting and the expression of SASP factors. Importantly, our results also presented the evidence showing that innate immune activation in progeria-aged myofibers is mostly induced by the cytosolic mtDNA leaked from damaged mitochondria, but not by cytosolic ncDNA from the nucleus. A potential mechanism for the process of cGAS-Sting-induced senescence-associated phenotype in progeria-aged myofibers is shown in Fig. 8.

Our recent study of Z24^−/−^ mice revealed the role of cytosolic ncDNA-induced cGAS-Sting innate immune response in mediating accelerated cellular senescence of muscle stem cells in progeria aging [24]. Muscle stem cells can actively replicate in number to meet the requirement for muscle regeneration, which confers progressively increased stress of DNA damage and telomere shortening. While myofiber is postmitotic multinucleated cell, and its ncDNA does not bear the replication stress and risk of telomere shortening. Therefore, the mechanism of cellular senescence induced by ncDNA damage and telomere shortening in muscle stem cells and many other mononuclear cells is supposed to be either waived or replaced by some different mechanism in myofibers. In contrast to muscle stem cells, myofibers are contractive and are highly active in cellular metabolism and energy production, which would require higher amount of actively functional mitochondria. It is possible that this high level of mitochondrial activity would consequently generate more mitochondrial damage. Our current results show that there is more oxidative damage of mtDNA in progeria aged myofibers, and more mtDNA can be leaked from damaged mitochondria to activate cGAS-Sting signaling, or can be easily accessed by cGAS protein through damaged mitochondria membrane, leading to excessive activation of innate immune response and senescence-associated phenotype. Damaged mitochondria are usually expected to be repaired by the fission/fusion process or cleaned by mitophagy [33]. However, our results show that, although mitophagy activation or initiation is elevated in myofibers of progeria mice, it seems that these myofibers have impaired capacity in effectively repair and clean these possibly damaged mitochondria, which may result in an accumulation of damaged mitochondria and dysfunctional mitophagy in the cells, as well as an excessive activation of cGAS-Sting signaling.

NF-κB signaling can be a part of innate immune reaction [35]. Our recent studies of Z24^−/−^ and ERCC1-/delta progeria mice revealed that NF-κB inactivation is an effective way in rescuing the senescent phenotypes of the mice [25, 26]. Based on our current results, it is possible that the results in our previous studies of progeria aging mice models can be further explained as, the inhibition of NF-κB actually reversed the senescence phenotypes of the mice by repressing the innate immune activation in various types of cells [25, 26].

Our result revealed a higher level of DNA damage (γ-H2AX+) in the mitochondria of Z24^−/−^ myofibers, but not in the nucleus of Z24^−/−^ myofibers (Fig. 1). But interestingly, the level of γ-H2AX protein at perinuclear areas of WT myofibers seems to be increased too (Fig. 1B), indicating a potentially higher level of damaged mitochondria and mtDNA at the location. Perinuclear mitochondria are commonly observed in various types of cells, exhibiting higher mobility, fission/fusion events and being critical in mitochondrial turnover [39]. Therefore it is possible that in WT myofibers, the mobility and fission/fusion events of mitochondria are still normal, and the damaged mitochondria are still able to move to perinuclear area to be repaired; while in Z24^−/−^ myofibers, the damaged mitochondria may not be able to move to perinuclear area smoothly to allow for the efficient repairing.

It is also interesting to notice in our results that, γ-H2AX is positive in many Z24^−/−^ MPCs but is completely lost in Z24^−/−^ myotubes. It is possible that DNA damage is more frequent in progeria-aged MPCs compared to myotubes, because MPCs are proliferative and require nucleus to divide, which may cause increased stress and damage to DNA. Also, when DNA in MPCs replicates, there is a chance of increased error or DNA damage too. While in the postmitotic myotubes, there is no cell or nuclear dividing, and DNA in the nucleus does not replicate; the nuclear structure does not need to be disturbed, and therefore DNA in the myofibers possibly gets less stress and damage. Also, the Z24^−/−^ MPCs with excessive DNA damage may have lost the myogenic capacity to form myotubes.

VDAC1 is the most abundant protein on the mitochondria outer membrane, and is a gatekeeper for the passages of metabolites, nucleotides, and ions. An interesting recent study showed that, N-terminal domain of VDAC1 interact with mtDNA and promote VDAC1 oligomerization and mtDNA release, and the VDAC oligomerization inhibitor VBIT4 decreases mtDNA release, IFN signaling and disease severity in a mouse model of systemic lupus erythematosus [28]. In our study, VBIT4 treatment of Z24^−/−^ myofibers in vitro was effective in inhibiting mtDNA-induced cGAS-Sting activation and expression of SASP factors, suggesting that VDAC1 can be a novel molecular target for improving progeria aging-associated defective phenotypes.

Our results indicate that, the increased mitophagy activation in progeria-aged myofibers should be both a direct result of mitochondria damages and a compensatory response for the decline in normal mitochondrial function. We observed increased mtDNA release into cytosol of Z24^−/−^ myofibers, which could be a result of increased mitochondria damage and membrane permeabilization mediated by VDAC1 oligomerization. Also, increased mitochondria damages in Z24^−/−^ myofibers can lead to mitophagy activation or initiation, in an effort to prevent mitochondrial apoptosis and cell death [40]. Here we did observe increased mitophagy activation or initiation in Z24^−/−^ myofibers, but the function of mitophagy seemed to be greatly compromised. Thus, more mitophagy activation or initiation may have been promoted in the myofibers as a compensatory response mechanism for increased mitophagy function. Meanwhile, the elevated accumulation of damaged mitochondria can directly induce further mitophagy activation or initiation. Therefore, the increased mitophagy activation or initiation in Z24^−/−^ myofibers should be a result of both mitochondria damage and a compensatory response.

In general, our current results revealed a novel mechanism for senescence-associated phenotypes of postmitotic myofiber in progeria-aged mice, which involves increased cGAS-Sting activation caused by mitochondria damage, VDAC1 oligomerization, and mtDNA release. Our current results verified a crucial role of mtDNA release and mitophagy dysfunction-mediated innate immune activation in mediating LMNA mutation-induced progeria phenotypes, which may help identifying a novel set of diagnostic markers and therapeutic targets for progeria aging.

### Supplementary information

Suppplemental data

Supplemental materials-Western blot original images

## Data Availability

The datasets generated during the current study are available from the corresponding author on reasonable request.
