# Supplementary material for: mtDNA release promotes cGAS-STING activation and accelerated aging of postmitotic muscle cells
Source: Cell Death Dis. 2024 Jul 23;15(7):523. doi: 10.1038/s41419-024-06863-8 (PMC11263593; doi:10.1038/s41419-024-06863-8)
Supplement: Supplementary file 1 — Suppplemental data [file 41419_2024_6863_MOESM1_ESM.docx]

**Supplemental Figures and legends:**


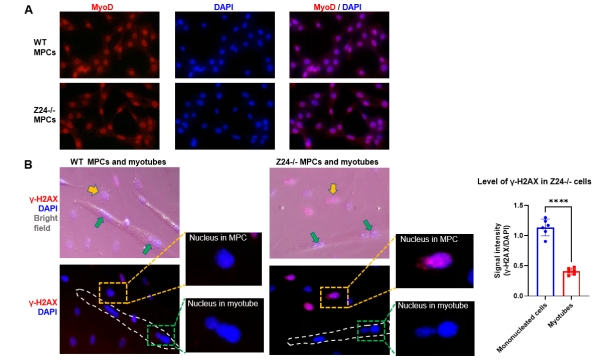


**Supplemental Figure 1: DNA damage is not higher in nucleus of Z24^-/-^ myofibers.**

**A.** WT-MPCs and Z24^-/-^ MPCs isolated from gastrocnemius muscles of mice (8-week old, female) were cultured *in vitro* and immunostained with antibody of MyoD, to verify the identify of MPCs. **B.** WT-MPCs and Z24^-/-^ MPCs were cultured in myogenic differentiation medium for 3 days to allow for myotube formation *in vitro*, and immunostaining of γ-H2AX was performed. The level of DNA damage in WT mononucleated cells, Z24^-/-^ mononucleated cells, WT myotubes and Z24^-/-^ myotubes is shown. Statistics of the γ-H2AX level in Z24^-/-^ mononucleated cells and Z24^-/-^ myotubes is shown. Data were from 3 groups of individual preparation of MPCs isolated from mice (8-week old, female) (biological replicates). And among each group of MPCs, cells from 2 individual wells were counted as technical replicates. Statistics are shown as mean values ± SD (N=6).


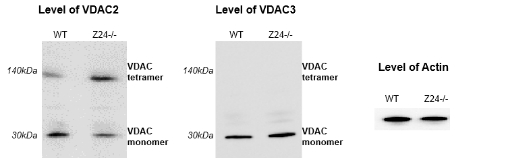


**Supplemental Figure 2: Western blot assay of VDAC2 and 3 in myofibers.**

Western blot assay was performed to check the potential oligomerization of VDAC2 and VDAC3 proteins in WT and Z24^-/-^ myofibers.


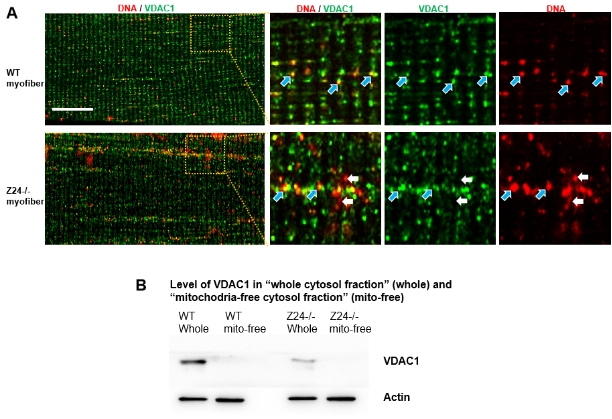


**Supplemental Figure 3: There is more mtDNA releasing from mitochondria into cytosol in Z24^-/-^ myofibers.**

**A.** Single myofibers isolated from FDB muscles of WT and Z24^-/-^ mice (5-month old, male) were cultured *in vitro* and were co-immunostained with antibodies to DNA and VDAC1, and imaged with Airyscan Fast Confocal Microscope. The localization of mtDNA and mitochondria is shown. In contract to regular mitochondrial localization of mtDNA (overlayed/colocalized with VDAC1 signal) in WT myofibers (blue arrows), some of mtDNA localized outside of mitochondria (not overlayed /colocalized with VDAC1 signal) in Z24^-/-^ myofibers (white arrows), especially at the area of mitochondria with higher VDAC1 signal (potentially increased VDAC1 expression and oligomerization). **B**. Western blot assay was performed with lysate of whole cytosol fraction and mitochondria-free cytosol fraction, to verify the purity of mitochondria-free cytosol fraction. VDAC1 protein is applied as a mitochondria-specific marker. Scale bar: 20μm.


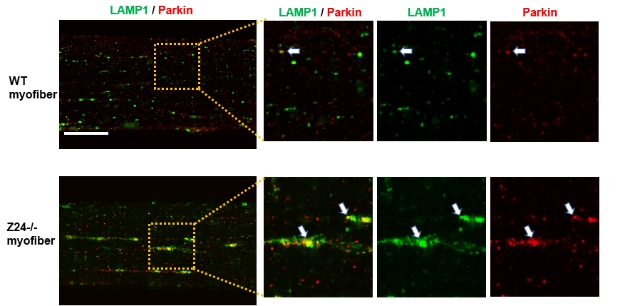


**Supplemental Figure 4: Z24^-/-^ myofibers develop higher level of mitophagy activation.**

Single myofibers isolated from FDB muscles of WT and Z24^-/-^ mice (5-month old, male) were cultured *in vitro* and co-immunostained for LAMP1 and Parkin, and imaged with Airyscan Fast Confocal Microscope. It shows increased level of LAMP1+, Parkin+ and LAMP1+/Parkin+ (white arrows) vesicles in Z24^-/-^ myofiber. Scale bar: 25μm.

**Supplemental Tables:**

| **Gene** | **Primer sequence** |
| --- | --- |
| GAPDH | Forward: TCCATGACAACTTTGGCATTG  Reverse: TCACGCCACAGCTTTCCA |
| CXCL1 | Forward: CTGGGATTCACCTCAAGAACATC  Reverse: CAGGGTCAAGGCAAGCCTC |
| MCP1 | Forward: TAAAAACCTGGATCGGAACCAAA  Reverse: GCATTAGCTTCAGATTTACGGGT |
| IL-1alpha | Forward: TCTCAGATTCACAACTGTTCGTG  Reverse: AGAAAATGAGGTCGGTCTCACTA |
| IL-1beta | Forward: GCAACTGTTCCTGAACTCAACT  Reverse: ATCTTTTGGGGTCCGTCAACT |
| IL-6 | Forward: CTGCAAGAGACTTCCATCCAG  Reverse: AGTGGTATAGACAGGTCTGTTGG |
| IFN-β | Forward: CAGCTCCAAGAAAGGACGAAC  Reverse: GGCAGTGTAACTCTTCTGCAT |
| TNF-α | Forward: CCTGTAGCCCACGTCGTAG  Reverse: GGGAGTAGACAAGGTACAACCC |
| IL-10 | Forward: ATTTGAATTCCCTGGGTGAGAAG  Reverse: CACAGGGGAGAAATCGATGACA |

**Supplemental Table 1: qRT-PCR Primer sequences**

| **Antibody** | **Vendor and catalog number** |
| --- | --- |
| cGAS | Cell Signaling 31659; Proteintech 29958-1-AP |
| Sting | Abcam 288157; Proteintech 19851-1-AP |
| p-TBK1 | Cell Signaling 5483 |
| TBK1 | Cell Signaling 3504 |
| LC3 | Abcam 48394 |
| p62 | Cell Signaling 39749 |
| VDAC1 | Abcam 306581; Santa Cruz 390996 |
| VDAC2 | Abcam 316107 |
| VDAC3 | Proteintech 55260-1-AP |
| γ-H2AX | Cell signaling 7631 |
| GAPDH | Santa Cruz 32233 |
| Actin | Santa Cruz 58673 |
| 8-OHdG | Santa Cruz 66036 |
| Collagen IV | Abcam 6586 |
| LAMP1 | Cell signaling 99437 |
| PINK1 | Santa Cruz 517353 |
| Parkin | Santa Cruz 32282 |
| DNA | Santa Cruz 58749 |
| Tom20 | Santa Cruz 17764 |
| p-p65 | Cell signaling 3033 |

**Supplemental Table 2: antibodies.**
