# Supplementary material for: mtDNA release promotes cGAS-STING activation and accelerated aging of postmitotic muscle cells
Source: Cell Death Dis. 2024 Jul 23;15(7):523. doi: 10.1038/s41419-024-06863-8 (PMC11263593; doi:10.1038/s41419-024-06863-8)

Figure 2C VDAC1

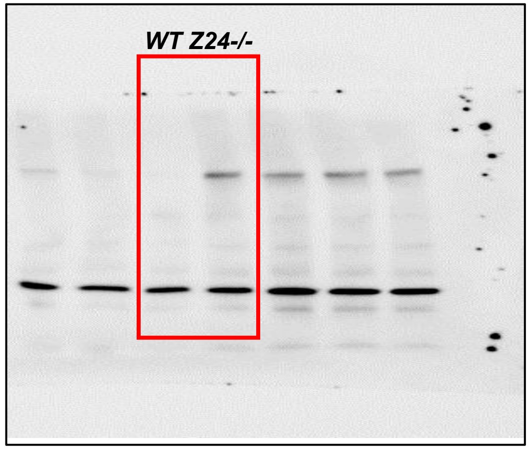

Figure 4C LC3

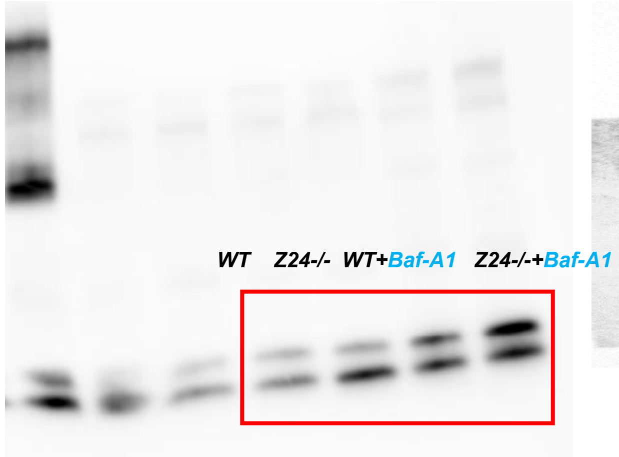

Figure 4C p62

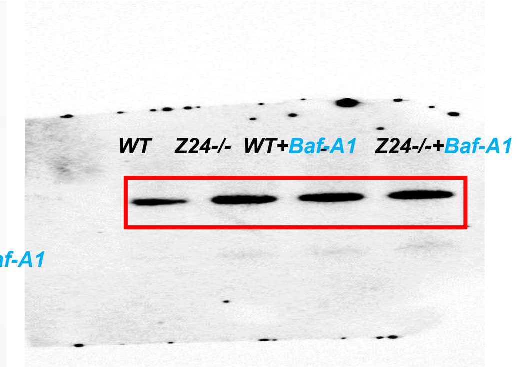

Figure 4C GADPH

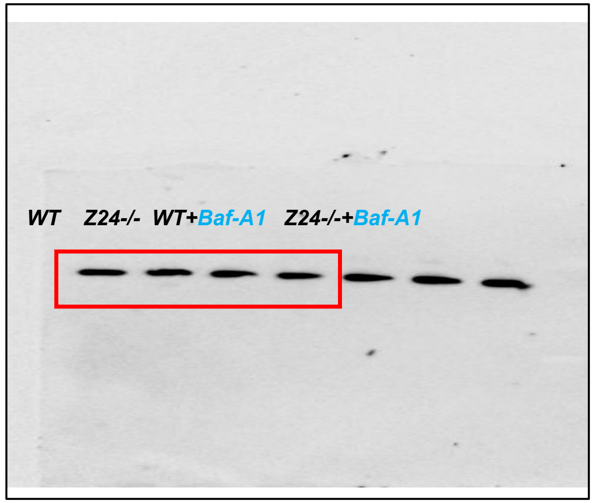

Figure 7A VDAC1

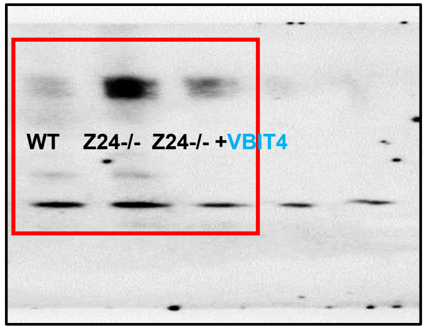

Figure 7A cGAS

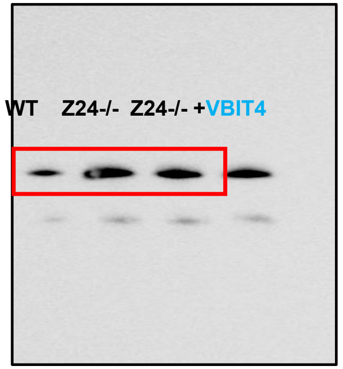

Figure 7A Sting

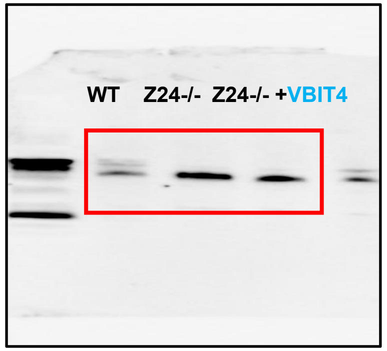

Figure 7A p-TBK1

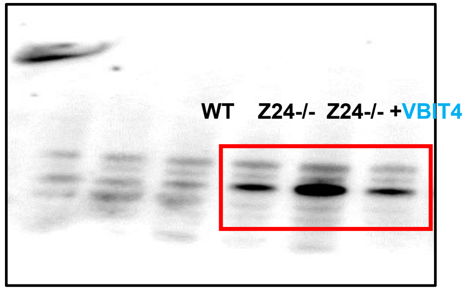

Figure 7A TBK1

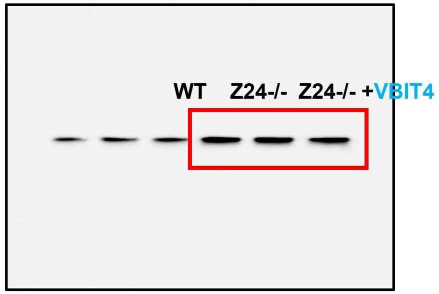

Figure 7A GADPH

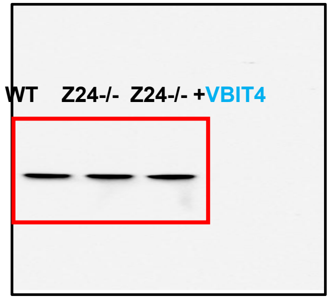

S Figure 2 VDAC2

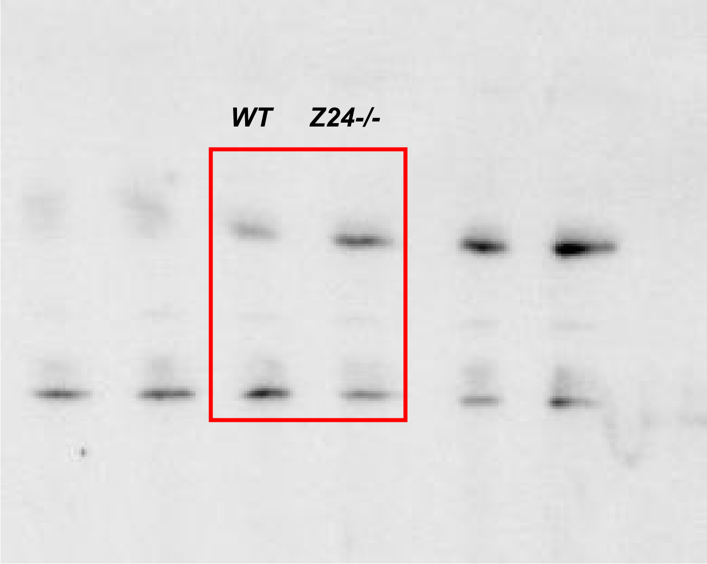

S Figure 2 VDAC3

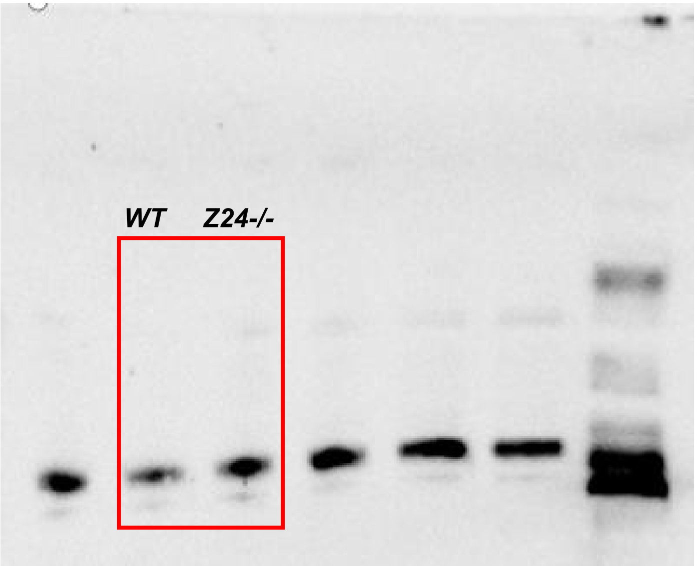

S Figure 2 actin

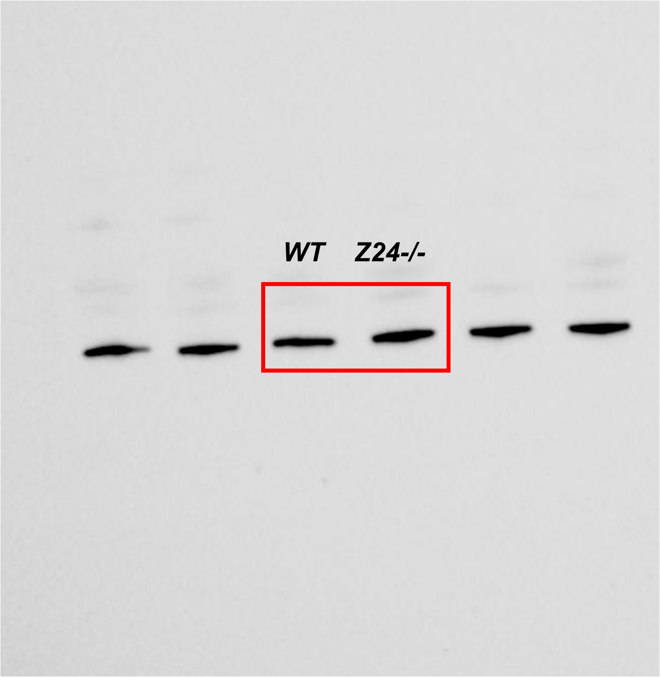

S Figure 3B VDAC1

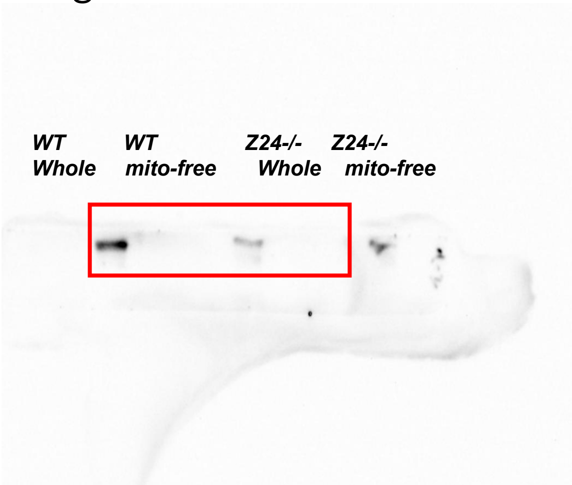

S Figure 3B actin

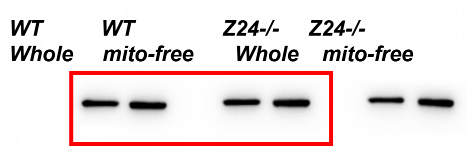

Supplement: Supplementary file 2 — Supplemental materials-Western blot original images [file 41419_2024_6863_MOESM2_ESM.pdf]
